# Genetic Variability of PRRSV Vaccine Strains Used in the National Eradication Programme, Hungary

**DOI:** 10.3390/vaccines9080849

**Published:** 2021-08-03

**Authors:** Ádám Bálint, Tamás Molnár, Sándor Kecskeméti, Gábor Kulcsár, Tibor Soós, Péter M. Szabó, Eszter Kaszab, Kinga Fornyos, Zoltán Zádori, Krisztián Bányai, István Szabó

**Affiliations:** 1 National Food Chain Safety Office Veterinary Diagnostic Directorate, H-1143 Budapest, Hungary; kecskemetis@nebih.gov.hu; 2 National PRRS Eradication Committee, H-1021 Budapest, Hungary; iszabodr@t-online.hu; 3 National Food Chain Safety Office Directorate of Veterinary Medicinal Products, H-1107 Budapest, Hungary; kulcsarg@nebih.gov.hu (G.K.); soost@nebih.gov.hu (T.S.); 4 Molecular Medicine Research Group, Hungarian Academy of Sciences and Semmelweis University, H-1085 Budapest, Hungary; dr.szabo.peter@gmail.com; 5 Institute for Veterinary Medical Research, H-1143 Budapest, Hungary; kaszab.eszter@atk.hu (E.K.); zadori.zoltan@atk.hu (Z.Z.); banyai.krisztian@atk.hu (K.B.); 6 M. A. H. Food Controll Ltd., H-1211 Budapest, Hungary; fornyos.kinga@vet-controll.hu; 7 Department of Pharmacology and Toxicology, University of Veterinary Medicine, H-1078 Budapest, Hungary

**Keywords:** porcine reproductive and respiratory syndrome virus, molecular epidemiology, phylogenetic network

## Abstract

Porcine reproductive and respiratory syndrome (PRRS) is a globally spread, highly infectious viral disease. Live, attenuated vaccines against PRRS virus (PRRSV) decrease virus excretion and evoke protective immunity reducing the economic damage caused by the disease. In a longitudinal molecular epidemiological study accompanying ongoing national eradication programme we evaluated the suitability of PRRSV ORF5 and ORF7 sequences to identify possible field strains of vaccine-origin. In total, 2342 ORF5 sequences and 478 ORF7 sequences were analysed. Vaccine strains were identified by sequence identity values and phylogenetic network analysis. Strains that shared greater than 98% nucleotide identity within ORF5 and/or ORF7 were considered to have originated from vaccine. A total of 882 (37.6%) ORF5 and 88 (18.4%) ORF7 sequences met these criteria. In detail, 618, 179 and 35 ORF5 and 51, 29 and 8 ORF7 sequences were related to Porcilis PRRS vaccine, Unistrain PRRS vaccine, and ReproCyc PRRS EU vaccine, respectively. Data showed that the Porcilis vaccine was genetically more stable. Whereas, the variability of the Unistrain and the ReproCyc strains was significantly higher. Given that ORF7 shares, in some instances, complete identity between a particular vaccine strain and some historic variants of field PRRSV strains, care must be taken when evaluating vaccine relatedness of a field isolate based on the ORF7. On the contrary, ORF5 sequences were more suitable to predict the vaccine origin making a distinction more robustly between field and vaccine strains. We conclude that ORF5 based molecular epidemiological studies support more efficiently the ongoing PRRS eradication programmes. The conclusions presented in this large-scale PRRS molecular epidemiological study provides a framework for future eradication programmes planned in other countries.

## 1. Introduction

Porcine reproductive and respiratory syndrome (PRRS) is an infectious disease with worldwide distribution that leads currently to the highest economic loss in pig production [1,2,3]. The disease is caused by an enveloped single-stranded RNA virus (PRRS virus, PRRSV) belonging to the Arterivirus genus within the Arteriviridae family of the Nidovirales order. Based on their 30–45% genetic distance, PRRSV strains can be classified into two distinct species Betaarterivirus suid-1 (PRRSV-1) and Betaarterivirus suid-2 (PRRSV-2) [4]. The disease was first described by Keffaber in the United States in 1989 [5], then emerged in Canada, Germany and the Netherlands [6]. In Hungary, seropositivity was first observed in the mid-1990s among imported animals, and the virus was isolated in 1996 [7,8].

Vaccines play an extremely important role in the control of various infectious diseases, including PRRS [9]. In order to meet the requirements of specific protection against infectious diseases, vaccines must be safe, efficacious and of appropriate quality. Consequently, the fulfilment of the same requirements is also an essential condition for the authorization of a vaccine.

It is important to be aware that PRRSV is one of the most variable RNA viruses [10]. It is also known that virulent PRRSVs can only elicit a relatively weak humoral and cellular immune response [11,12]. Attenuated PRRS vaccines are primarily able to induce a relatively good immune response against genetically homologous strains, they are capable of mitigating economic losses, but have only partial protection against heterologous infectious strains [9,13,14]. Although higher genetic similarity in many cases does correlate with improved vaccinal protection [15]. It is also known that following immunisation with live vaccines, pigs develop viraemia and shed the vaccine virus for several weeks [9,16,17], which can be transmitted directly or indirectly to susceptible unvaccinated animals. It has also been demonstrated that vaccine strains in live virus vaccines are able to recombine with virulent strains [14,18,19,20]. It is also known that relatively small changes in the genome of the vaccine strain can lead to reversion to virulent phenotype [21]. The specific characteristics of live attenuated virus vaccines used in an eradication programme may influence the cost of the eradication, and the efficiency of the animal health epidemiological activities.

In general, live, attenuated vaccines against PRRSV are capable of reducing PRRS virus excretion in infected stocks, the clinical symptoms of the virus (respiratory and reproductive lesions of young, and adult animals, respectively), and hence, the economic damage caused by the disease. However, they are unable to prevent and inhibit the infection.

Unistrain PRRS (earlier Amervac PRRS vaccine) and Porcilis PRRS vaccines had been widely used in pigs in Hungary more than a decade before the PRRS eradication programme was introduced in 2014. In Hungary, the first authorisation to market the Porcilis PRRS vaccine was given on 6 May 2002, while the Unistrain PRRS vaccine (Amervac PRRS vaccine) was approved on 28 April 2004. In this early pre-eradication period, vaccination was used to reduce the economic losses caused by the disease.

Reprocyc PRRS EU and Ingelvac PRRS FLEX EU vaccines were authorised in Hungary on 24 February 2015, following the introduction of the eradication programme. Both vaccines contain strain PRRSV 94881.

The date of first authorisation of Suvaxyn PRRS vaccine manufactured by Zoetis is on 24 August 2017. Due to the significant progress to this date in the PRRS eradication programme for large pig populations, the market share of this vaccine has not reached those of the previously registered vaccines.

Roughly 29% of the large breeding herds in Hungary used Porcilis PRRS vaccine, 25% used Unistrain PRRS and 9% used Reprocyc PRRS. The rest (37%) used an inactivated vaccine during the eradication (Table 1).

Given the characteristics of PRRSV, it was considered important to monitor the epidemiological observations of the extensive vaccinations carried out during the nationwide eradication and the data provided on the shedding, stability and possible modification of the vaccine strains. In a framework that produced over 2000 PRRSV-specific sequences over a 10–year-period of virus monitoring, we performed this nested study and raised the question whether the PRRSV ORF5 and/or ORF7 sequences are suitable to determine the possible vaccine-origin of the identified strain in a farm under control and active virus surveillance.

## 2. Materials and Methods

### 2.1. Swine Herds

The study was carried out in Hungary and involved pig farms, which were/are in the process of PRRS eradication. Only the farms implementing an immunization program using a live virus vaccine were included in the present study. The examined swine farms were mainly large scale farrow-to-finish type of 300–2500 sows with different status of infection and vaccination. They represented 13 out of 19 counties of Hungary.

### 2.2. Vaccines

Vaccines developed from live attenuated virus strains used in the national PRRS eradication programme were as follows: Vaccine A, Porcilis PRRS vaccine (manufacturer: MSD Animal Health, Madison, NJ, US); Vaccine B, Unistrain PRRS vaccine (formerly: Amervac), (manufacturer: Laboratorios Hipra, S.A., Amer, Spain); Vaccine C, Reprocyc PRRS EU vaccine (manufacturer: Boehringer Ingelheim Vetmedica GmbH, Ingelheim am Rhein, Germany); Vaccine D, Ingelvac PRRSFLEX EU vaccine (manufacturer: Boehringer Ingelheim Vetmedica GmBH, Ingelheim am Rhein, Germany); Vaccine E, Suvaxyn PRRS MLV (manufacturer: Zoetis Belgium SA) (Table 1).

### 2.3. Sample Collection

The infected farms, based on their eradication programme, were routinely sampled on a weekly/monthly basis to monitor the herd status by blood and organs (e.g., lungs, lymph nodes, foetuses) for PRRS testing. Blood samples were obtained from suckling, weaning, pre-fattening and fattening pigs, as well as breeding animals (boars, sows, gilts).

Besides regular monitoring, samples were taken from animals showing reproductive or respiratory disorders (organ samples from weaning, prefattening, fattening animals and aborted foetuses).

The other group of samples originated from imported prefatteners, 48 h after arriving in Hungary. These animals were vaccinated in the country of origin.

### 2.4. Diagnostic Examinations

Serological (ELISA) and virological (PCR) tests were performed on blood samples from different age groups to determine the time of infection. This process was used to build up a farm-specific eradication protocol, including not only the vaccination regime, but also internal biosecurity measures.

PRRSV ELISA tests from pig sera were carried out applying the INgezim PRRS Universal ELISA Kit (Ingenasa, Madrid, Spain) according to the recommendations of the manufacturer.

RNA from serum samples was extracted with the KingFisher Flex system (ThermoFisher, Waltham, Ma, USA) using MagAttract 96 cador Pathogen Kit. PCR was performed in Rotor-Gene Q (Qiagen) real-time PCR machine using the virotype PRRSV TR-PCR Kit (Qiagen) according to the manufacturer’s instructions.

Samples positive by PCR were subjected to sequencing. The viral ORF5 was sequenced preferably, but in case of failure, the viral ORF7 was also sequenced. Sequencing was performed with the Sanger method using BigDye 3.1 kit (Applied Biosystems, Foster City, CA, USA) on ABI 3500 sequencer (Applied Biosystems). Chromatograms were analysed and edited manually using the BioEdit software version 7.2, available at http://www.mbio.ncsu.edu/BioEdit/bioedit.html [22].

### 2.5. Sequence Analysis

In this study, 2342 PRRSV ORF5 sequences (606 nt) and 478 PRRSV ORF7 sequences (387 nt) were analysed. Out of the ORF5 sequences, 69 were found in GenBank and published in international literature, while the other were the result of assays in Hungarian laboratories. Out of the domestic sequences, 118 were generated before 2013, 47 in 2014, and 2060 sequences were generated between 2015 and 2019. From the ORF7 sequences, 36 were present in Genbank, 42 in 2013, 27 in 2014, and 373 in 2015 and 2019 in Hungary. A total of 832 ORF5 and 88 ORF7 sequences were deposited in GenBank (accession numbers: MT628907–MT629731).

Sequence analysis was performed using the “similarity network” diagram [23] to identify the closest, most similar sequences to the virus in each vaccine. Subsequently, the percentile similarity of these sequences to ORF5 and ORF7 was evaluated relative to ORF5 and ORF7 of the PRRSV strain of the live virus vaccine tested in GenBank., From 2014 onwards, epidemiological data (veterinary information on the origin of the sample, immunisation etc.) were assigned to each Hungarian sequence/sample.

Using this practical and easy-to-follow enriched minimum spanning similarity network application for improved representation of phylogenetic relations among viral strains, we eliminated the necessity of applying a pre-defined, arbitrary cut-off or computationally extensive algorithms. The network-based visualization allowed processing and visualizing a large amount of vaccine and wild-type PRRSV, and helped identify the potential connections between different viral sequences that support data-driven decisions in the eradication programmes of the different farms.

## 3. Results

### 3.1. Porcilis PRRS Vaccine

#### 3.1.1. Sequence Analysis of ORF5

**Figure 1:**
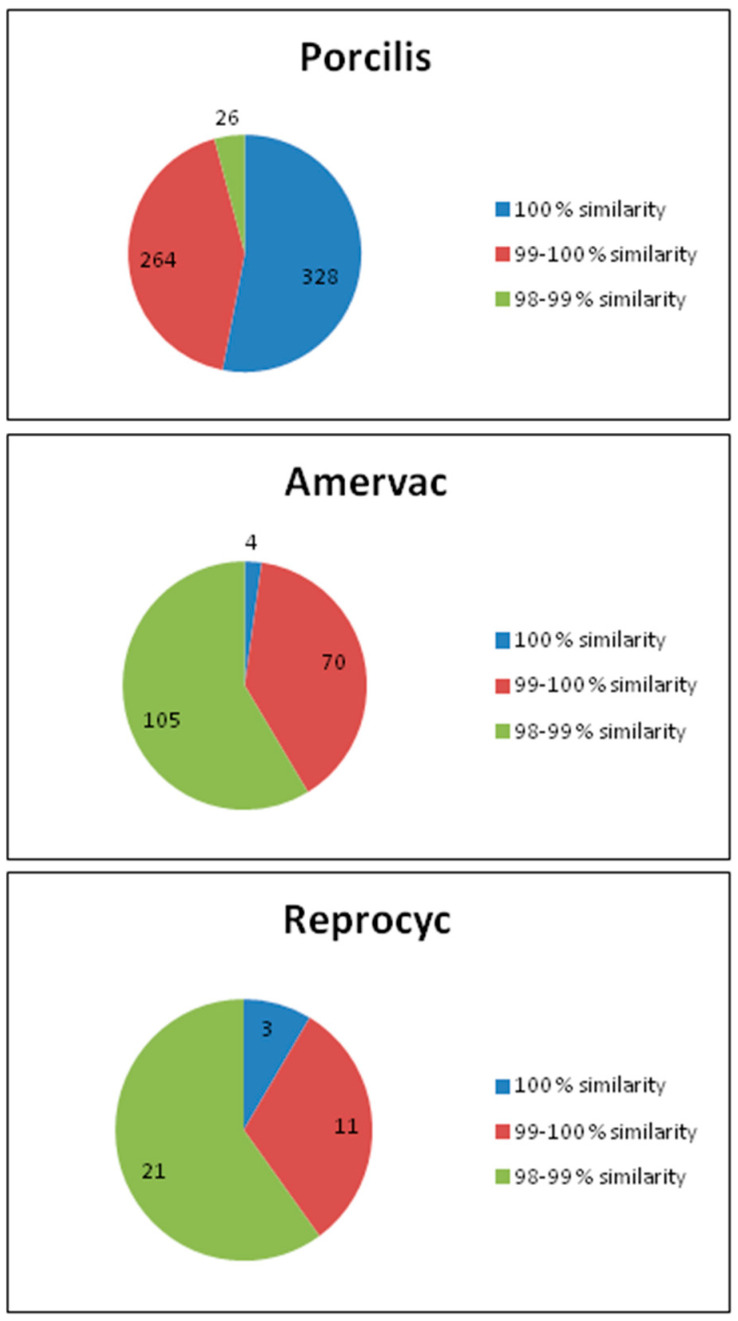
Distribution and sequence similarity of vaccine-related ORF5 sequences.

Of the 2342 PRRSV ORF5 sequences tested, 618 were found to share at least 98% similarity to the strain in the Porcilis PRRS vaccine. Of these sequences, 328 (53.1%) were 100% identical to the ORF5 sequence of the vaccine strain (including the Porcilis PRRS vaccine strain), 264 (42.7%) shared a similarity between 99.1 and 99.9%, and 26 (4.2%) had 98–99% similarity. Of note is that the Lelystad strain belongs to this group (Figure 1). 

In breeding herds from which the ORF5 sequence was at least 99% identical to the Porcilis PRRS vaccine virus, only this vaccine was used for immunisation against PRRS. Examining the origin of the sequences, we found that 260 of the 264 sequences that were detected in sera of vaccinated and unvaccinated suckling piglets, vaccinated piglets at battery age, and vaccinated during quarantine period (twice with 2–3 weeks interval) had similarity more than 99% to the original vaccine strain. This value was 98.61% for 3 sequences.

In the case of those fattening units filling with nursery pigs of Hungarian origin, 104 sequences were determined. Of these, 102 sequences showed greater than 99%, while two showed 98–99% similarity with the ORF5 region of the Porcilis PRRS vaccine. Porcilis PRRS immunisation was performed for each of these flocks.

In our experience, when sentinel animals (*n* = 19) were housed among fattening pigs previously immunised with Porcilis vaccine the isolated viruses shared 99–100% similarity within the ORF5 region of the vaccine virus. Similarly, when an unvaccinated fattening group was kept in the same airspace with breeding sows that were immunised with Porcilis PRRS vaccine and provided PCR positive samples, the identified virus strain showed 100% nucleotide identity with the vaccine virus.

**Figure 2:**
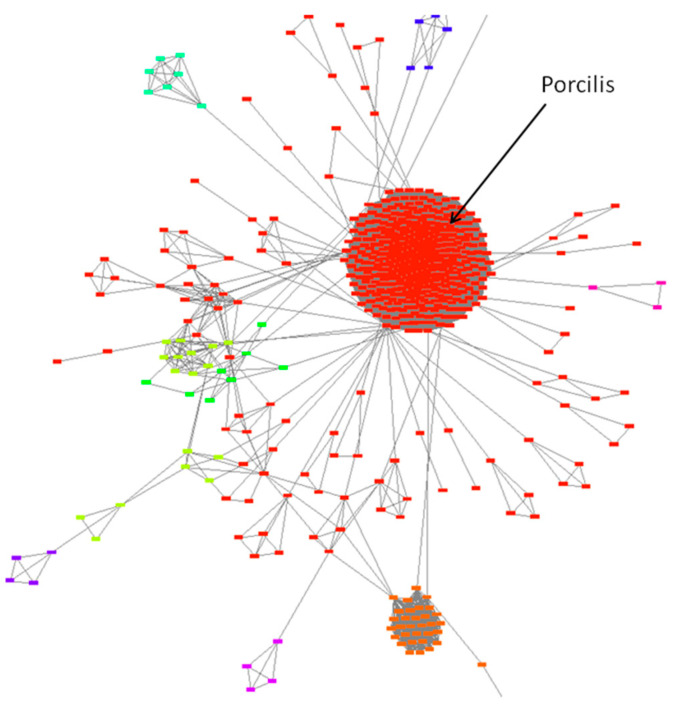
Genetic stability of Porcilis PRRSV vaccine using the similarity network approach.

The high genetic stability of Porcilis PRRSV vaccine was confirmed by applying the similarity network approach, and no herd-specific lineages were identified (Figure 2).

#### 3.1.2. Sequence Analysis of ORF7 

The live attenuated PRRS DV virus strain of Porcilis PRRS vaccine has an ORF7 sequence 100% identical to the Lelystad PRRSV ORF7 region, based on international literature. The Lelystad virus is a wild type reference strain of PRRSV1, which was first detected in European PRRS epidemics [24].

Therefore, it should be carefully evaluated whether a PRRSV ORF7 sequence detected in a herd may be derived from the use of a Porcilis PRRS vaccine prior to sampling. According to the manufacturer, the vaccine virus may be excreted (shed) from vaccinated animals for up to 5 weeks after vaccination, but this may take longer in a larger population.

**Figure 3:**
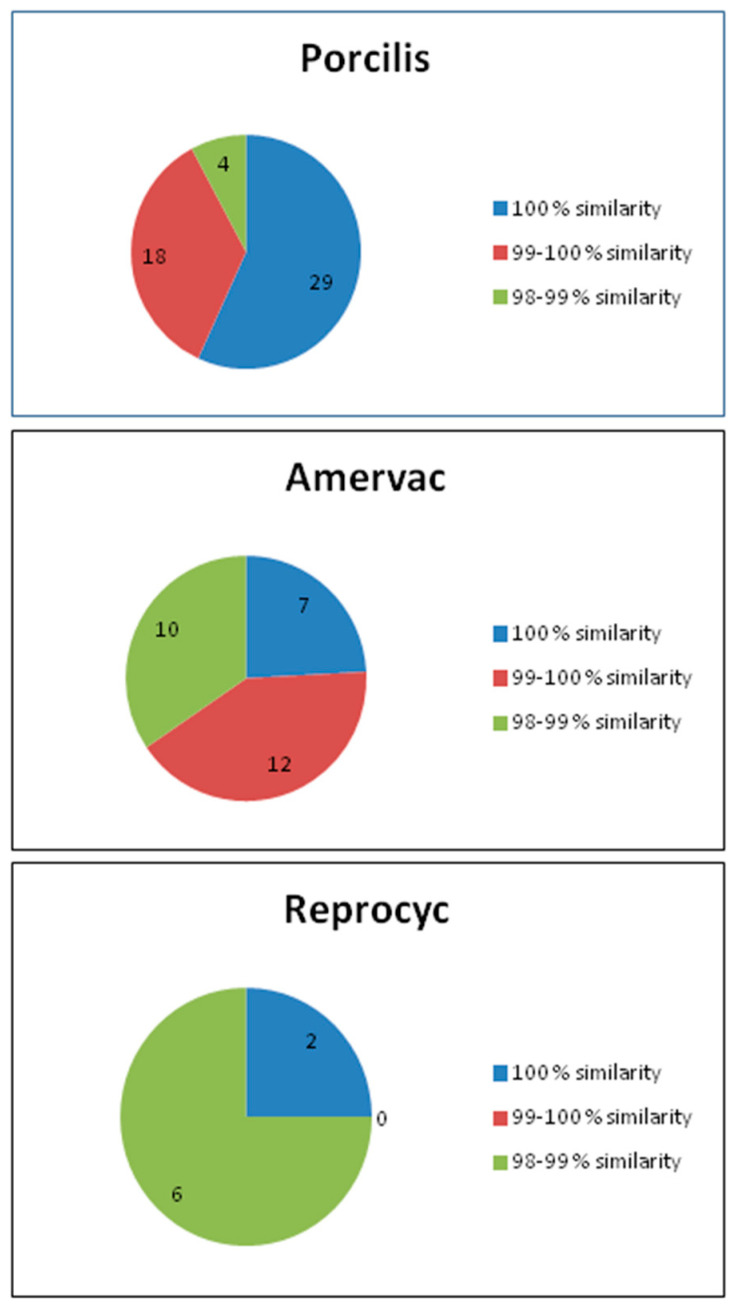
Distribution and sequence similarity of vaccine-related ORF7 sequences.

In Hungarian pig herds controlled by the Porcilis PRRS vaccine, 29 (56.9%) of the PRRSV ORF7 sequences were 100% identical to the Porcilis PRRS vaccine strain or the Lelystad ORF7 sequence. Eighteen (35.3%) and four (7.8%) sequences shared 99.1–99.9% and 98–99% similarity, respectively, with the vaccine strain. All 51 PRRSV ORF7 sequences were obtained from herds that had been vaccinated with Porcilis PRRS (Figure 3).

### 3.2. Unistrain PRRS Vaccine (Former Amervac)

#### 3.2.1. Sequence Analysis of ORF5

Among the 2342 PRRSV ORF5 sequences, 179 were found to be at least 98% similar to the strain in the Unistrain PRRS vaccine. From these sequences, four (including the Amervac and Unistrain vaccine strains and two international sequences: DQ345725.1 (MLV vaccine), and JQ040769.1 (clone AN0708EU_1) were 100% identical with the ORF5 sequence of the vaccine strain. 99.1 to 99.9% similarity was seen in 70 (39.1%) sequences, and less than 99% but more than 98% was observed in 105 (58.7%) sequences (Figure 1). None of the sequences detected in Hungarian swine herds were 100% identical with the ORF5 sequence of the PRRSV strain in the Unistrain PRRS vaccine (GenBank acc. no.: MK134483).

In breeding herds where samples showed the highest ORF5 sequence identity (at least 99%) with the Unistrain PRRS vaccine virus, this vaccine was used in all cases for immunisation against PRRS. Similar associations were seen in fattening farms where animals that shed PRRSV strains with at least 99% sequence identity to the homologous vaccine strain originated from breeding farms having used Unistrain PRRS in the vaccination program. Furthermore, the Unistrain PRRS vaccine was used in all breeding and fattening farms where the isolated strain shared 98.1–98.9% similarity with the vaccine strain (Figure 1).

Further data analysis revealed that 79 sequences were detected in blood samples collected from nursery pigs 3–10 weeks post vaccination. Of these, 48 sequences shared <99% similarity to the Amervac vaccine virus strain. The remainders showed >99% similarity. A total of 30 sequences were from unvaccinated suckling piglets or pre-fatteners whose sows were immunised during their pregnancy. In 26 of these cases, the ORF5 region was <99% similar to the Amervac vaccine virus strain, in the remainder the similarity value fell between 99.07% and 99.54%.

**Figure 4:**
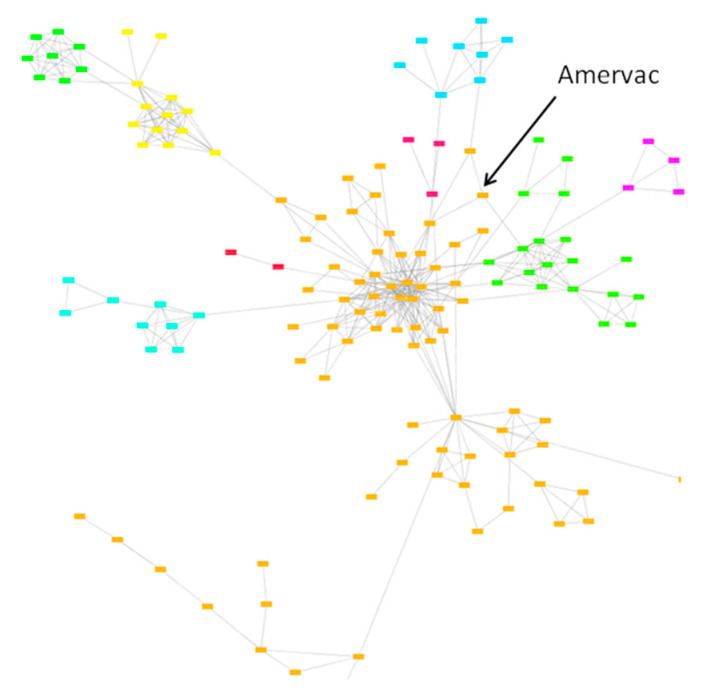
Genetic stability of Unistrain PRRSV vaccine using the similarity network approach.

The genetic heterogeneity of the Unistrain PRRS vaccine was confirmed by using the similarity network method. Sequences originating from various farms using the vaccine are indicated by different colours. Data suggest that the emergence and evolution of farm-specific lineages originated from the vaccine strain; these lineages shared different degree of similarity compared with the original vaccine strain (Figure 4).

#### 3.2.2. Sequence Analysis of ORF7

The analysis of the ORF7 gene identified 29 sequences that showed >98% similarity to the Unistrain PRRS virus strain. Seven sequences–including the Amervac vaccine strain–were 100% identical to the ORF7 sequence of Amervac PRRSV. Epidemiological data showed that these seven samples were collected from herds used Amervac vaccine in the breeding stock or in the fattening unit (*n* = 4); the remainder of herds reported no use of Amervac (*n* = 1), or reported to use another vaccine (*n* = 1), Pyrsvac (manufactured by LABORATORIOS SYVA), a live, attenuated PRRS vaccine not registered in Hungary. In 12 cases, the ORF7 sequence identity values determined from field samples fell between 99% and 100% when compared with the ORF7 of Amervac PRRSV. In eight cases, the Amervac vaccine was used in the respective age group herd, while a pig of 16 weeks of age has not been vaccinated, but the sow population was vaccinated with Unistrain 10 days prior to weaning. Out of the remaining three sequences, two originated from import of pre-fatteners, and one was a Spanish PRRSV (65_2_Spain_1991) in Genbank.

In ten cases, the sequences showed greater than 98%, but less than 99% identity with the ORF7 sequence of Amervac PRRSV. In nine of these, Amervac vaccine was used in the herd, and in one case no information was available.

We investigated the epizootological circumstances on three large scale farms using Amervac immunisation, as part of the animal health technology. In one of these farms, which was farrow-to-wean type, Amervac vaccination was used three times after PRRSV infection on all pigs in the herd. PRRSV ORF7 detected in the sera of newborn piglets showed a high degree of similarity to Amervac (but less than 100%). In an all-in-all-out fattening herd where animals arrived from the above mentioned farm, and were immunised at the age of 3 weeks with Amervac on the breeding unit, a sequence similar to Amervac (greater than 99% but less than 100%) was determined. In the third case, in a farrow-to-finish farm, where sows were vaccinated with Unistrain during the pregnancy, sequences showing the same degree of similarity were detected in non-vacinated pigs from 8 weeks of age. The epidemiological origin of the three sequences described above confirmed 99.53% similarity to the ORF7 sequence of the Amervac vaccine strain. In these cases, it is also likely that the samples represented a live attenuated vaccine virus strain used for immunisation, or their slightly different variants within a relatively short period of time.

### 3.3. ReproCyc PRRS EU Vaccine (and Its Non Adjuvanted Variant Ingelvac PRRSFLEX EU Vaccine)

#### 3.3.1. Sequence Analysis of ORF5

In our study, 35 ORF5 sequences showed at least 98% similarity to the virus strain in the ReproCyc PRRS EU vaccine. From these sequences, three (8.6%),–including the Reprocyc vaccine strain–were completely identical to the ORF5 sequence of the vaccine strain. For 11 (31.4%) and 21 (60%) sequences, similarity was between 99.1 and 99.9%, and less than 99% but more than 98%, respectively.

During the detailed epidemiological analysis, we found that two Hungarian sequences were 100% identical to the PRRSV strain in the Reprocyc PRRS vaccine. These two sequences were detected in pigs raised on a fattening farm where the animals were not immunised with the PRRS FLEX vaccine, but in the building where these animals were kept, the previously resident herd was immunised with the above mentioned vaccine.

Additional sequences closely related (99.15% to 99.76%) to the vaccine strain were detected in this farm from animals housed simultaneously in other buildings Another further six closely related sequences were identified in animals that originated from breeding farms where the Reprocyc vaccine was used to implement their eradication programme.

In the 98.1 to 98.9% similarity group, there was one fattening farm for which no information was available on the use of Reprocyc PRRS vaccine (Figure 1).

**Figure 5:**
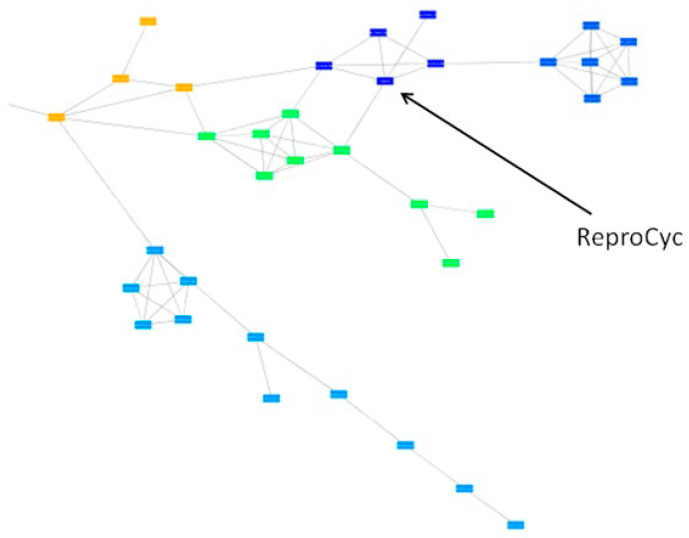
Genetic stability of ReproCyc PRRSV vaccine using the similarity network approach.

The genetic heterogeneity of the Reprocyc or PRRSFLEX PRRS vaccine was confirmed by using the similarity network method. Again, farm-specific lineages developed over time that showed various degree of similarity compared with the original vaccine strain (Figure 5).

#### 3.3.2. Sequence Analysis of ORF7

Concerning the analysis of the ORF7 gene only eight sequences were found, which were >98% similar to the ORF7 region of the Reprocyc PRRS vaccine strain.

These include the Ingelvac_ORF7 and Reprocyc_ORF7 sequences, which are derived from, and are identical with the vaccine. Another two sequences, Amervac and Pyrsvac were 98.13% similar to ORF7 of the Reprocyc vaccine.

Only one of the remaining sequences was related to the use of the Reprocyc vaccine, while the other sequences were related to the use of the Amervac vaccine (Figure 3).

## 4. Discussion

Since the detection of PRRSV infection in Hungary, farmers have primarily protected their livestock against the economic damage of the disease by vaccination. However, Hungary was the first country in the world to implement a national eradication programme for PRRS infected swine herds in an organised manner. The legal basis for the eradication scheme was laid down after consultation with the major stakeholders in the pig sector, breeders, integrators, practice and science [25].

The principle for the eradication of infected swine herds is the strict keeping of the external and internal disease control standards of the farms, laboratory monitoring of different age groups of the herds and immunisation against PRRS (including pregnant and non-pregnant breeding sows, replacing gilts, breeding boars, piglets during lactation, pre-fatteners, and fatteners). The main purposes of immunisation are as follows: (a) Development of homogeneous immune status of infected breeding stock and prevention of intrauterine and neonatal infections; (b) promoting the virus-free raising of progeny of breeding sows; (c) production of PRRS virus-free gilts from their own breeding stock, or from outer source, thereby replacing the infected breeding stock with virus-free sows [26].

The identification of PRRSV ORF5 and ORF7 sequences provides extremely important information on the spreading of PRRSV, the determination of the possibility of infection and the ways of spreading the epidemic. The visualization of the similarity among the sequences and the visualization of epidemiological relationships significantly enhance the possibility for practitioners to analyse epidemiological processes through virological (molecular biology based) results [23].

The evaluation of sequence data is very important for the investigation of the PRRS epidemic, for the detection of virus spread between individual farms and within the herd. In order to assess the specific disease control and the current status of the eradication, it is necessary to determine safely whether the PRRS PCR positivity in each age group is caused by the wild type virus or by the applied live attenuated vaccine strain [27].

In this study, 2342 PRRSV ORF5 and 478 ORF7 sequences were analysed. In relation to the viral strains in live attenuated PRRS vaccines, we found that: (i) 832 of the ORF5 sequences (35.5%) showed greater than 98% homology to any vaccine strain used in the particular pig farm; (ii) among the 832 sequences showing similarity to the vaccines, 618 (74.3%) were Porcilis, 179 (21.5%) were Unistrain (formerly Amervac) and 35 (4.2%) were considered to be derived from Reprocyc; (iii) regarding the vaccine strain associated sequence similarity data, the majority of Porcilis strains showed absolute (100%) homology or very high sequence identity (i.e., 99.1–99.9%), while a minor fraction of Porcilis derived sequences showed lower sequence identity (98–99%). Of interest, the Unistrain derived sequences and the Reprocyc derived sequences showed an inverse pattern of homology. Based on these findings Porcilis seems to maintain its stability, while the variability of the Unistrain and the Reprocyc strains was significantly higher.

It is also important to note that whenever sentinel animals were housed among fattening pigs previously raised in the colony and previously immunised with Porcilis vaccine, all sequences obtained from the sentinel animals showed greater than 99% similarity to the ORF5 region of the vaccine virus. An unvaccinated fattener group kept in the same airspace with breeding gilts immunised with Porcilis PRRS vaccine has been found to be PCR positive, and the PCR product from their sera 100% matched to the vaccine virus (the fatteners were not immunised). For some fattener herds, it is likely that the import of prefattening animals or their sows were immunised at the place of origin with the Porcilis PRRS vaccine. Of the 74 sequences derived from these cases, only two were less than 99% similar to the ORF5 region of the vaccine strain.

Based on our investigations, we have the following conclusions:

(1) The ORF5 region of the strain of the Porcilis PRRS vaccine showed that irrespective of the way the vaccine virus entering the body of the pig (vaccination or “infection” with after vaccination shed virus), it is consistently stable and such genetic change does not occur during the period of entry that would interfere with virus vaccine sequencing. The PRRSV strain found in the Porcilis PRRS vaccine does not seem to be prone to significant genetic changes. If the PRRSV ORF5 or ORF7 sequence is at least 98% similar to the Porcilis PRRSV strain, it is safe to say that Porcilis PRRSV strain was detected (Figure 1 and Figure 3).

In the case of the Unistrain PRRS (Amervac) vaccine, genetic changes occur relatively rapidly, often to a considerable extent, after administration of vaccine (Figure 1 and Figure 3).

The relatively limited data available for the Reprocyc PRRS vaccine does not allow a similar conclusion to be made.

(2) Comparison of the ORF5 and ORF7 regions of PRRSV with the ORF5 and ORF7 regions of the three live vaccines can be estimated that if they are ≥99% similar, it is likely that the sequence detected in the sample is a consequence of a direct application or indirect spread. However, care should be taken with regard to the ORF7 section of the Porcilis PRRS vaccine strain, which is 100% identical to the ORF7 section of the Lelystad PRRS strain.

We found that a 100% match between the PRRSV ORF7 sequence detected in the blood samples of a given swine herd and the ORF7 sequence of the Amervac PRRSV strain means in the epidemiological sense that the sequence of the live attenuated PRRSV ORF7 used for vaccination has been detected.

(3) Based on sequence analysis, the sequencing of the PRRSV ORF5 section alone is sufficient in most cases to determine the PRRSV sequence identity. With care, useful information is also obtained by analyzing the PRRSV ORF7 sequence.
(4) For spreading the infections among herds, epidemiological investigation is greatly facilitated by the sequencing of PRRSV ORF5 and ORF7. During the epidemiological investigation it is important to know the origin of the herd tested and the knowledge of the immunisation applied in the surrounding farms. Knowledge of vaccine(s) used for immunisation against PRRS over several years in the investigated population is also essential. It is also indispensable to know the age of the sampled animals, the type of production and the time of arrival at the farm. In the eradicating process, when using live attenuated virus vaccines, the PCR positivity is very important to determine whether it is caused by the wild type virus or the vaccine virus.
(5) In relation to sequence distribution along the stages of the eradication programme, as the eradication process approached the final stage, more and more pig farms became free from the disease, and stopped vaccination. Therefore, the number of sequences of vaccine origin decreased. In contrast to the wild-type viruses where viral evolution, and emergence of new variants occurred along the long-term eradication process, only limited farm-specific vaccine sequence evolution was observed.
(6) Several PRRSV ORF5 and ORF7 sequences may be present at the same time in a single herd. Knowledge of a sufficient number of well-characterised PRRSV ORF5 and ORF7 se-quences available over the years is a pre-requisite for an adequately performed epide-miological investigation. It is necessary to evaluate as many PRRSV ORF5 and ORF7 sequences as possible in an infected herd from several animal groups and from differ-ent age groups. The use of a similarity network method can provide reliable and easy-to-understand information for sequencing. In this way, it is possible to determine with sufficient certainty the origin of the infection and to detect any further infections as soon as possible. In similarity network representation, the Porcilis strains are or-ganised into a single node confirming the genetic stability of this vaccine strain. In contrast, the Amervac and Reprocyc strains do not form a node but a dense network, and develop farm-specific minor lineages. These data can serve as useful information that may contribute to further PRRSV vaccine development.

## Figures and Tables

**Table 1:** Data of the vaccine strains used in Hungary.

Vaccine	Marketing Authorisation Holder	% of Applying Farms
Porcilis	MSD	29%
Progressis *	Ceva-Phylaxia (formerly Merial)	37%
Reprocyc	Boehringer Ingelheim	9%
Amervac, Unistrain	Hipra	25%

* Progressis is an inactivated vaccine.

## Data Availability

Sequence data were deposited in GenBank (acc.no., MT628907-MT629730).
